# Effect of Alloying Elements on the Short-Range Orders and Atomic Diffusion Behavior of Liquid Al−9Si Cast Alloys

**DOI:** 10.3390/ma16206768

**Published:** 2023-10-19

**Authors:** Xunming Zhu, Dan Liu, Jian Wang, Candong Chen, Xinxin Li, Li Wang, Mingxu Wang

**Affiliations:** 1 School of Mechanical, Electrical & Information Engineering, Shandong University, Weihai 264200, China; xunming.zhu@wfjt.com (X.Z.); wanglihxf@sdu.edu.cn (L.W.); 2 Weihai Wanfeng Magnesium Industry Science and Technology Development Co., Ltd., Weihai 264200, China; dan.liu@wfjt.com (D.L.); jian.wang@wfjt.com (J.W.); candong.chen@wfjt.com (C.C.); 3 School of Materials Science and Engineering, Liaocheng University, Liaocheng 252000, China; lixinxinlcu@163.com; 4 Weihai Institute of Industrial Technology, Shandong University, Weihai 264200, China

**Keywords:** AIMD, Al−Si alloy, liquid structure, diffusion coefficient

## Abstract

To investigate the influence of alloying elements (Zn, Mg, and Cu) on the structural and dynamical properties of liquid Al−9Si alloy, we conducted ab initio molecular dynamics (AIMD) simulations. Our results indicate that the structure of Al−Si−M ternary alloys is determined with a combination of atomic radii and mixing enthalpy, while the dynamic properties are primarily influenced by electronic structure of the alloying elements. Specifically, the addition of Cu promotes the formation of Al−Cu short-range order (SRO), while Zn has a higher propensity for Zn−Zn SRO. The Al−Cu SRO in liquid alloy may serve as the precursor for the Al_2_Cu reinforcing phase in Al−Si−Cu alloys. Upon the addition of Mg, a greater number of relatively stable perfect and distorted icosahedral structures, as well as hcp and bcc ordered structures with lower energies, are observed. Additionally, the presence of Mg leads to a reduction in the atomic diffusion rates of Al and Si, while Cu and Zn exhibit complex diffusion behavior influenced by the presence of Si atoms.

## 1. Introduction

Al−Si casting alloys are a group of materials widely used in the automotive, aerospace, and general engineering industries for various applications. Al−Si casting alloys offer a range of benefits, including excellent castability, good corrosion resistance, high strength-to-weight ratio, and good thermal conductivity. These characteristics make them a preferred choice for components that require lightweight design, high strength, and good heat dissipation. The alloys’ combination of lightweight design, strength, and good thermal management makes them particularly valuable in the automotive industry, where reducing weight and improving fuel efficiency are priorities [1].

Effects of alloying elements and other variables on microstructure and mechanical properties of Al−Si alloys have been widely investigated [2,3,4,5,6]. Previous research has indicated that Cu and Mg contribute to the strengthening of the Al−7%Si cast alloys, while Fe predominantly impairs their elongation [7]. The analysis of microstructure shows that an increase in Cu content within the alloys leads to higher quantities of intermetallic compounds and an elevated concentration of Cu in the *α*-Al matrix. Additionally, the porosity level remains unaffected, while the tensile strength improves at the expense of ductility [8]. By adding Co and Ni in a hypoeutectic Al–Si alloy, the tensile strength was improved up to 230 °C [9]. The presence of higher Cu and Ni contents led to enhanced tensile properties following T5 treatment and over-aging at 350 °C [5]. Research shows that alloying elements, especially Mg and Cu, are important means to improve the performance of Al−Si alloys [10,11]. Meanwhile, Zn has a very similar atomic radius and electron structure to Cu but can hardly be used as an alloying element to enhance the strength of Al−Si alloys. The existing studies mainly focus on the strengthening phases formed by alloying elements or their role in grain refinement. The local structure and behavior of these alloying elements in the molten state are rarely investigated due to experimental limitations. Therefore, it is interesting to study the structure and effects of these three elements in Al−Si alloys to provide insights for the selection of alloying elements.

The aim of this study is to investigate the structures of liquid Al−9Si alloy using ab initio molecular dynamic (AIMD) simulation. The impact of introducing small amounts of Cu, Mg, and Zn on the structures and fluidity of the Al−Si molten alloy is investigated by analyzing changes in chemical and topological short-range order (SRO), as well as self-diffusion coefficients in relation to different alloying elements. The simulation work is conducted to provide theoretical analysis of macroscopic changes at the atomic level, which is difficult to achieve through experiments.

## 2. Materials and Methods

This study was based on a new generation of high as-cast strength die-cast Al−9Si−based alloy [12]. In the case of the binary Al−9Si alloy, a supercell with periodic boundary conditions encompassed 200 atoms. Then, using five Zn, Mg, and Cu atoms, some of the Al atoms were replaced to form three alloys: Al−9Si−5M (M = Cu, Mg, Si). For the ternary alloys, a simulation involving 200 atoms was conducted to ensure an accurate and reliable statistical result. To establish the initial configurations, atoms were randomly stacked according to the defined concentration. Adjustments were made to the positions of atoms that were too close to one another in order to achieve realistic distances. Prior to data collection, the size of the supercell was modified to maintain the external pressure within ±1.5 Kbar, thereby causing negligible volume changes [13,14].

The simulations were performed using the Vienna ab initio simulation package (VASP) [15]. Interactions between ions and electrons were described using the projector augmented-wave method [16], and the electronic exchange and correlation were calculated by employing generalized gradient approximations [17]. Sampling of the supercell Brillouin zone was carried out solely at the Г point. In a canonical ensemble (NVT), the number of particles, volume, and temperature remained constant. Temperature control was achieved using a Nosé thermostat [18] with a frequency of 52 ps. The simulations were conducted at a temperature of 955 K to ensure that the alloys were completely in a liquid state. Configurations were saved at intervals of 3 fs, resulting in a collection of 6000 configurations for analysis over a period of 18 ps (Table 1).

**Figure 1:**
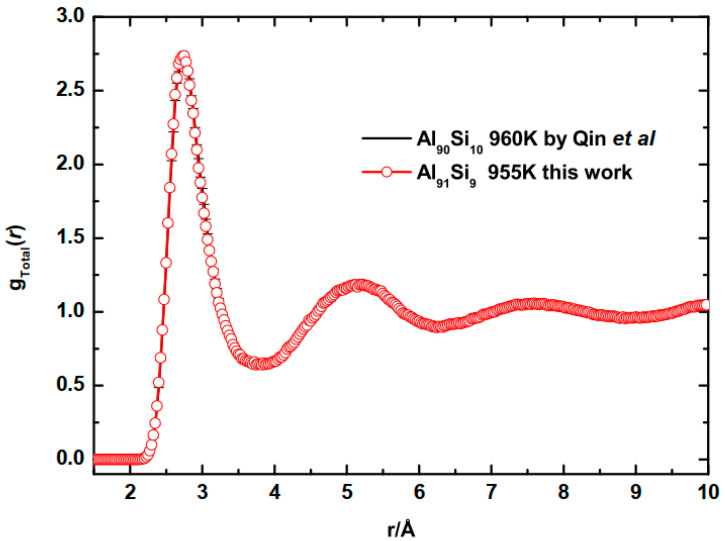
Comparison of the calculated $g_{Total}\left(r\right)$ of liquid Al−9Si alloy at 955 K and that of Al−10Si at 960 K by Qin et al. [19].

To validate the model, we compared the calculated $g_{Total}\left(r\right)$ of liquid Al−9Si alloy at 955 K and of Al−10Si at 960 K described by Qin et al. [19], as shown in Figure 1. It is observed that both the position and height of all peaks fit very well with that of previous work. Furthermore, the model of Qin et al. has been validated by comparing with experimental result [20]. The definition of *g*(*r*) is given in Section 3.1.

## 3. Results and Discussions

### 3.1. Pair Distribution Function

The pair distribution function (PDF) is a commonly used tool to describe the local structure of liquid and amorphous materials. The Faber–Ziman PDF is defined as [21]:

(1) $$g_{\alpha \beta }\left(r\right)=\frac{L^{3}}{N_{\alpha }N_{\beta }}\langle \sum_{i=1}^{N_{\alpha }}N_{i\beta }\left(r\right)\rangle /4\pi r^{2}\Delta r$$

**Figure 2:**
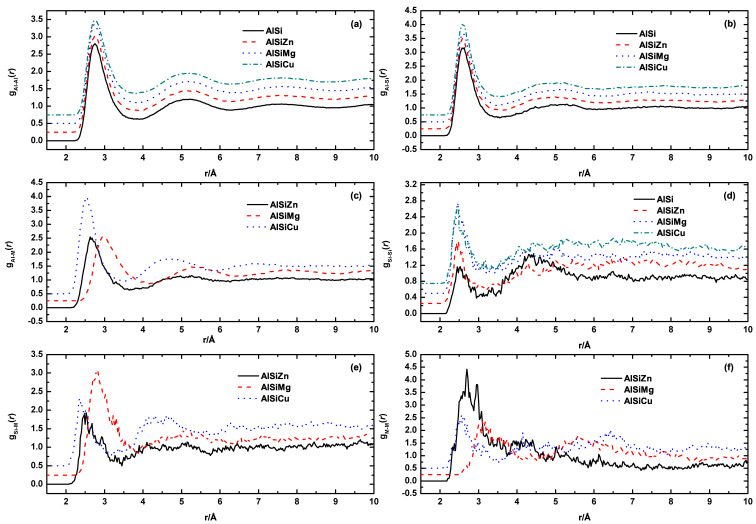
Partial pair distribution functions of Al−9Si, Al−9Si−5Mg, Al−9Si−5Cu, and Al−9Si−5Zn from (**a**–**f**): $g_{\mathrm{AlAl}}\left(r\right)$, $g_{\mathrm{AlSi}}\left(r\right)$, $g_{\mathrm{AlM}}\left(r\right)$, $g_{\mathrm{SiSi}}\left(r\right)$, $g_{\mathrm{SiM}}\left(r\right)$, and $g_{\mathrm{MM}}\left(r\right)$, respectively.

The parameters within the equation are defined as follows: L represents the length of the supercell, $N_{\alpha }$ and $N_{\beta }$ indicate the quantities of *α* and *β* atoms, respectively, and *N_iβ_* represents the count of *β* atoms surrounding the *i*th *α* atom within the spherical shell ranging from *r* to *r* + Δ*r*. In Figure 2, the six partial PDFs of Al−9Si and Al−9Si−5M alloys (M = Mg, Cu, Zn) are presented. It is worth noting that Al is the predominant element in all of these liquid alloys. Consequently, the influence of the alloying elements on the *g*_AlAl_(*r*) and *g*_AlSi_(*r*) function is negligible, as depicted in Figure 2a,b, respectively. The different positions of the first peaks of *g*_AlM_(*r*) function, as shown in Figure 2c, apparently result from the different atomic radii of Cu(1.28 Å), Zn(1.34 Å), and Mg(1.60 Å). According to the intensity of the three *g*_AlM_(*r*) curves, which represents the probability of finding M atoms around Al atoms at r position, Cu atoms are more likely to appear in the first neighbor shell around Al when compared with Zn and Mg atoms. Although there is a significant difference in atomic radii, the probability of Mg and Zn atoms appearing in the first coordination shell of Al atoms is almost the same. In liquid metals, the size of atoms plays an important role in determining the coordination number and the formation of bonds. The results of the three *g*_AlM_(*r*) curves indicate that atomic size is not the primary determining factor for the structure of alloying elements around Al atoms. Other factors such as mixing enthalpy may also play important roles. The electronic structures and atomic radii of Cu (1s² 2s² 2p⁶ 3s² 3p⁶ 4s¹ 3d¹⁰) and Zn (1s² 2s² 2p⁶ 3s² 3p⁶ 4s² 3d¹⁰) are very similar, yet their mixing enthalpy with Al are opposite. Al−Cu alloy has a negative mixing enthalpy (−1 kJ/mol), whereas the mixing enthalpy for Al−Zn is positive (1 kJ/mol) [22]. This implies that Cu atoms have a higher probability of being surrounded by Al atoms.

The *g*_AlSi_(*r*) curves indicate that in binary Al−Si alloys, Si atoms appear with nearly equal probability in the first and second coordination shells. However, with the addition of alloying elements, the alloying atoms replace the Si atoms in the second coordination shell around Al atoms. In other words, in ternary Al−Si−M alloys, Si atoms around Al atoms are only distributed in the first coordination shell. Figure 2f shows that, compared to Cu and Mg, Zn atoms have a higher tendency to bond with similar atoms. We believe this is also related to the positive mixing enthalpy between Al and Zn.

By considering the concentration c_i_, the chemical short-range order (SRO) can be obtained from *g_αβ_*(*r*) [23]:

(2) $$g_{cc}\left(r\right)=c_{\alpha }c_{\beta }\left[g_{\alpha \alpha }\left(r\right)+g_{\beta \beta }\left(r\right)-2g_{\alpha \beta }\left(r\right)\right]$$

**Figure 3:**
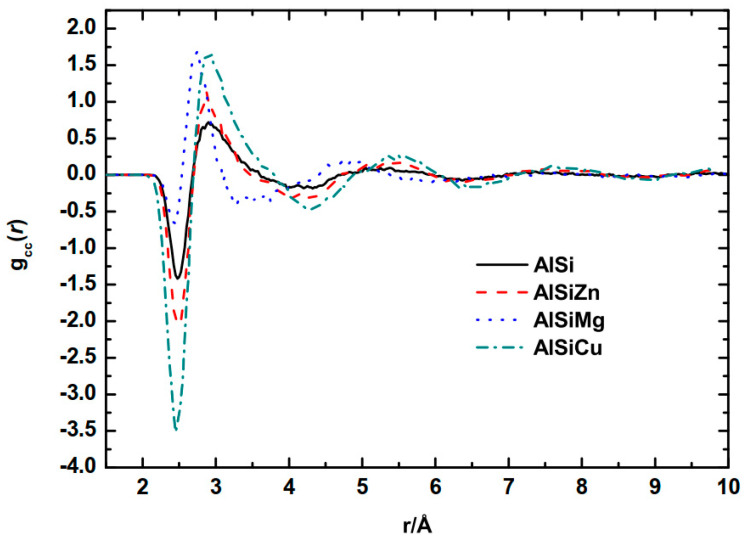
Concentration correlation *g*(*r*) function of Al−9Si−5M (M = Zn, Mg, Cu).

It can be observed that positive and negative peaks will arise to indicate preferences between like or unlike atoms, respectively. Figure 3 presents a comparison of $g_{cc}\left(r\right)$ for Al−9Si alloy and the three ternary melts. The most prominent negative peaks occur at ~2.45 Å for all four alloys. This value is significantly smaller than the Al−Si atomic distance of approximately 2.60 Å. Due to the superposition of the Al−M negative peak and Si−M negative peak, the position of the Al−Si negative peak is shifted. Overall, compared to Mg and Zn, Cu is more likely to form short-range chemical order around Al and Si atoms, which is consistent with Figure 2c,e. In the Al−Si−Mg ternary alloy, the probability of bonding between like atoms is much higher than that of unlike atoms. Moreover, with the addition of alloying elements, the ordered structure in the melt extends from the first coordination layer to approximately 6 Å, compared to the Al−Si binary alloy. This difference in ordered structure is not solely caused by the atomic radius effects.

### 3.2. Coordination Number

In order to quantitatively analyze the chemical SRO, we calculated the partial coordination number (CN) of species *β* around *α* by integrating the product of the number density of *β* (represented as *ρ_β_*) and the corresponding *g_αβ_*(*r*) function within the first coordination shell [24].

(3) $$N_{\alpha \beta }=\int_{0}^{r_{min}}4\pi r^{2}\rho_{\beta }g_{\alpha \beta }\left(r\right)dr$$

The minimum cutoff distance, *r_min_*, is determined as the distance after the first peak. The partial CNs and the constitutional proportion (designated *P_αβ_*) for each species are presented in Table 2. The overall $P_{\alpha \beta }$ is defined as the sum of the CNs around each species: $P_{\alpha \beta }=N_{\alpha \beta }/\sum^{\;}N_{\alpha i}\left(i=\alpha ,\;\beta ,\;\gamma \ldots \right)$. It is observed that the $P_{\alpha \beta }$ value for Al−9Si closely matches its nominal proportion, indicating the absence of chemical SRO in this melt. It is worth noting that the largest deviation from the nominal composition in Al−9Si−5Zn is observed for *P*_ZnZn_, indicating that Zn atoms in the alloy have a greater tendency to bond with each other. This is due to the positive mixing enthalpy between Al and Zn, as well as the relatively small atomic radius of Zn, making it more prone to aggregation with like atoms. In addition, in Al−9Si−5Cu, both *P*_CuSi_ and *P*_SiCu_ are significantly lower than their nominal compositions, while *P*_SiAl_ and *P*_CuAl_ are notably higher than their nominal compositions. Looking at the mixing enthalpy, the mixing enthalpy between Si and Cu (−19 kJ/mol) is much smaller than the mixing enthalpy between Cu and Al (−1 kJ/mol). Therefore, we believe that this is primarily due to the atomic radius effect. Since Al atoms have a relatively larger atomic radius, they provide more space to accommodate Cu and Si atoms.

### 3.3. Bonding Pair Analysis

**Figure 4:**
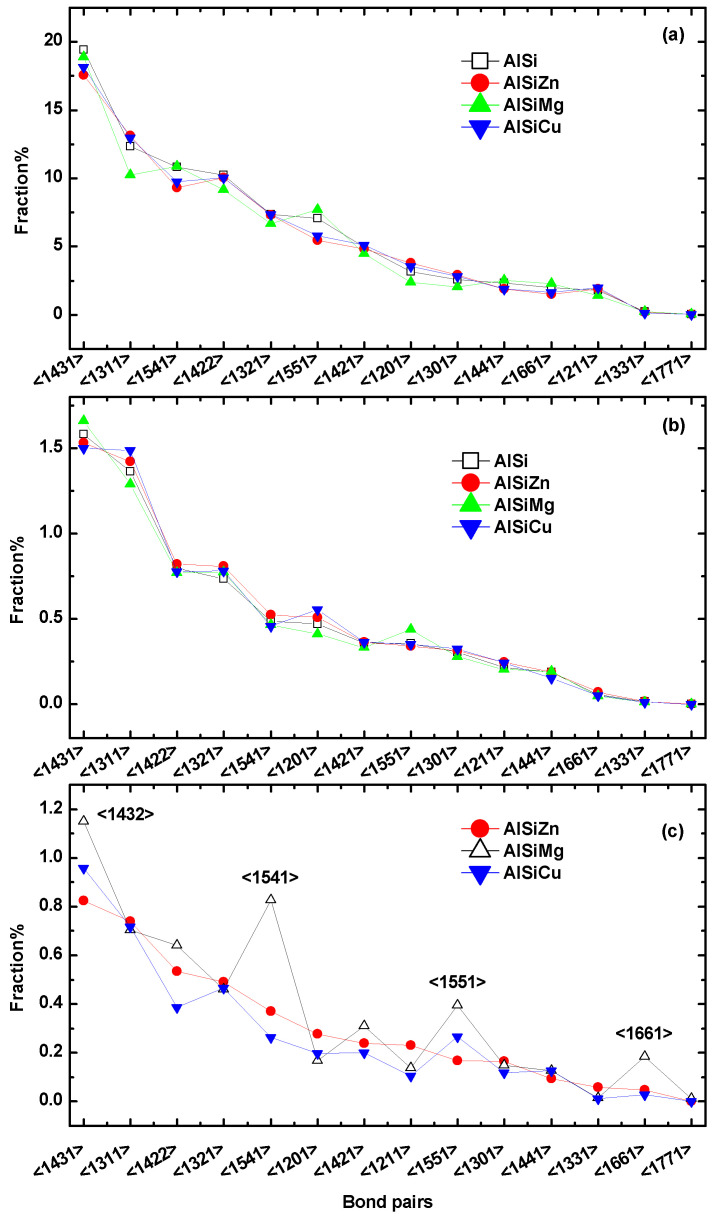
The distribution of BP around (**a**) Al, (**b**) Si, and (**c**) M (M = Cu, Mg, Zn) for different melts; the BP index is arranged in descending order of their abundance in Al−9Si for (**a**,**b**), and in Al−9Si−5Zn for (**c**).

In order to provide a comprehensive understanding of the SRO, we employed the proposed bonding pair (BP) analysis [25,26]. Figure 4 illustrates the distribution of 14 representative bond pairs around Al, Si, and M (M = Cu, Mg, Zn) for different melts. For Figure 4a,b, we firstly list the 14 most prevalent bond pairs in Al−9Si in descending order of abundance. And then the proportions of the same bond pairs for the other three ternary alloys are also listed in the same order as Al−9Si. This method allows for a comparative statistical analysis of Voronoi polyhedra, which represents the topological structure, in several liquid alloys. Figure 4c follows the same method but is based on Al−9Si−5Zn as a reference.

It is worth noting that the BP of Al and Si in different melts are structurally consistent, indicating that the addition of the third element has a minimal impact on the topological SRO of Al and Si. By analyzing the BP of the M element, it can be observed that the distribution of BP in Cu and Zn is relatively similar, while there is a significant difference in Mg. This indicates that the topological coordination is primarily influenced by atomic radii. Compared to Al−9Si−5Zn and Al−9Si−5Cu, the Al−9Si−5Mg alloy exhibits a higher proportion of <1432>, <1541>, <1551>, and <1661> structural motifs associated with Mg. Among the four, <1432>, <1541>, and <1551> are typically associated with the formation of perfect or distorted icosahedral ordering, and 1661 pairs are commonly observed in hcp and bcc structures [27,28]. According to Qi et al. [29], the population of bonds is influenced by their energy and distortion. Therefore, it implies that in the ternary alloy, there are more energetically favorable perfect and distorted icosahedral structures surrounding Mg, as well as a more ordered hcp and bcc structures.

### 3.4. Diffusion Coefficient

**Figure 5:**
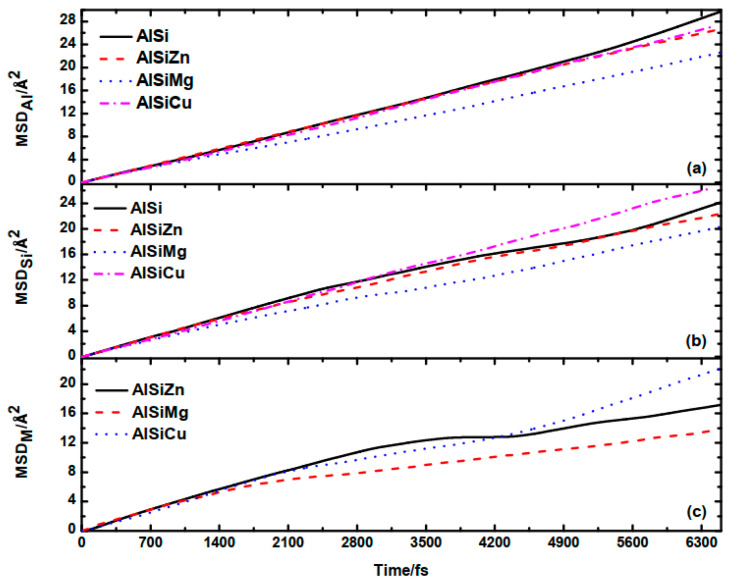
The MSD of Al (**a**), Si (**b**), and M (M = Zn, Mg, Cu) (**c**).

In order to investigate the influence of alloying elements on the diffusivity of the Al−9Si melt, we calculate the self-diffusion coefficient using the mean square displacement (MSD) for each species:

(4) $$\Delta r_{\alpha }{\left(t\right)}^{2}=\frac{1}{N_{\alpha }}\langle \sum_{i=1}^{N_{\alpha }}{\left|r_{\alpha i}\left(t+t_{0}\right)-r_{\alpha i}\left(t_{0}\right)\right|}^{2}\rangle$$

where the sum is taken over all *N_α_* atoms of species *α*, *t*_0_ represents an arbitrary time origin, and the angular brackets denote a thermal average or an average over time origins. Typically, the diffusion coefficient *D_α_* of species *α* has a linear relationship with the MSD according to the following:

(5) $$\langle \Delta r_{\alpha }{\left(t\right)}^{2}\rangle \to 6D_{\alpha }t+B_{\alpha }$$

where *B_α_* is a constant term. Figure 5 depicts the time dependence of the MSD for each species.

From Figure 5, it can be observed that the addition of alloying elements Zn and Cu has almost no impact on the atomic diffusion rates of Al and Si. However, after the addition of Mg, the atomic diffusion rates of Al and Si noticeably decrease, indicating a decrease in their mobility or fluidity.

According to the research findings of Zhu et al. [14], the presence of Si in aluminum alloys absorbs the outer valence electrons of the metallic elements in the alloy, thereby influencing the atomic diffusion behavior of these metallic elements. This leads to a transition from linear diffusion to parabolic or more complex motion patterns. For further study, we calculated the distribution of valence electrons in the four alloys using the Bader program. The valence electrons of each element are listed in Table 3. It is evident that after adding Mg atoms, most of the valence electrons around Mg are acquired by Si. Compared to transition metal elements, i.e., Cu and Zn, the valence electrons of Mg atoms are more reactive and more likely to interact with the valence electrons of Si. The interaction between these valence electrons suppresses the diffusion of the atoms.

The diffusion coefficients *D_α_* for each species are listed in Table 4. For M element, *D_α_* is approximated through linear fitting of the MSD with respect to time after 2.1 ps, where a significant change in the trend of MSD occurs. As observed, the *D*_Al_ and *D*_Si_ in Al−9Si−5Mg decreases by 23.9% and 14.0%, respectively, after the addition of Mg, highlighting the significance of the alloying element.

## 4. Conclusions

Our findings reveal that the structure of liquid Al−Si−M ternary alloys depends on both atomic radii and mixing enthalpy, and the dynamic property is mainly related to electronic structures of alloying elements. Al−9Si−5Cu and Al−9Si−5Zn have similar topological structures, but they exhibit significant differences in their chemical SRO. In other words, the variations in the effects of Cu and Zn on Al−9Si alloys are attributed to differences in their chemical SRO. The addition of Cu leads to the formation of more Al−Cu SRO, while Zn has a greater propensity to form Zn−Zn SRO. This could also be the reason why Al_2_Cu reinforcing phase forms more easily during the solidification process of Al−Si−Cu alloys. Most of the valence electrons around Mg are acquired by Si, and the interaction between these valence electrons suppresses the diffusion of the atoms. Furthermore, the interaction between Mg and Si provides clues for the formation of the Mg_2_Si strengthening phase in the Al−9Si−5Mg alloy.

## Figures and Tables

**Table 1:** Compositions, temperatures, number densities, and average external pressure of investigated liquid alloys.

Composition	Temperature (K)	Number Density (Å^−3^)	External Pressure (Kbar)
Al−9Si	955	0.052	1.22
Al−9Si−5Mg	955	0.051	−0.89
Al−9Si−5Cu	955	0.053	1.42
Al−9Si−5Zn	955	0.052	−0.55

**Table 2:** Partial CN and constitutional proportion of each species in the four liquid alloys.

Terms/*αβ*	Al−9Si		Al−9Si−5Zn		Al−9Si−5Mg		Al−9Si−5Cu	
	CN	*P_αβ_* (%)	CN	*P_αβ_* (%)	CN	*P_αβ_* (%)	CN	*P_αβ_* (%)
AlAl	10.94	92.37	10.07	87.34	10.16	86.54	10.25	87.84
AlSi	0.904	7.630	0.937	8.130	0.858	7.310	0.928	7.950
AlM	-	-	0.523	4.540	0.722	6.150	0.492	4.210
SiAl	9.137	97.41	8.951	93.88	8.203	88.36	8.869	94.51
SiSi	0.243	2.590	0.284	2.980	0.451	4.850	0.310	3.310
SiM	-	-	0.299	3.140	0.630	6.790	0.205	2.190
Mal	-	-	9.000	79.81	12.42	87.30	8.455	92.50
Msi	-	-	0.539	4.780	1.134	7.970	0.369	4.040
MM	-	-	1.738	15.41	0.672	4.720	0.316	3.460

**Table 3:** Valence electrons of Al, Si, and alloying elements Zn/Mg/Cu.

Elements	Al−9Si	Al−9Si−5Zn	Al−9Si−5Mg	Al−9Si−5Cu
Al	2.83	2.81	2.92	2.78
Si	5.67	5.37	5.54	5.42
M		12.88	0.68	12.20

**Table 4:** Self-diffusion coefficients (10^−9^ m^2^/s) of Al, Si, and alloying elements Zn/Mg/Cu.

Elements	Al−9Si	Al−9Si−5Zn	Al−9Si−5Mg	Al−9Si−5Cu
Al	4.51	4.13	3.43	4.28
Si	3.49	3.36	3.00	4.15
M		2.15	1.58	3.24

## Data Availability

All data included in this study are available upon request by contacting the corresponding author.
